# Skeletal Muscle Fibers Inspired Polymeric Actuator by Assembly of Triblock Polymers

**DOI:** 10.1002/advs.202105764

**Published:** 2022-03-06

**Authors:** Weijie Wang, Xian Xu, Caihong Zhang, Hao Huang, Liping Zhu, Kan Yue, Meifang Zhu, Shuguang Yang

**Affiliations:** ^1^ State Key Laboratory for Modification of Chemical Fibers and Polymer Materials Center for Advanced Low‐dimension Materials College of Materials Science and Engineering Donghua University Shanghai 201620 P. R. China; ^2^ South China Advanced Institute for Soft Mater Science and Technology School of Molecular Science and Engineering South China University of Technology Guangzhou 510640 P. R. China

**Keywords:** actuation, hydrogen‐bonding complexation, microphase separation, pH responsive, triblock copolymers

## Abstract

Inspired by the striated structure of skeletal muscle fibers, a polymeric actuator by assembling two symmetric triblock copolymers, namely, polystyrene‐*b*‐poly(acrylic acid)‐*b*‐polystyrene (SAS) and polystyrene‐*b*‐poly(ethylene oxide)‐*b*‐polystyrene (SES) is developed. Owing to the microphase separation of the triblock copolymers and hydrogen‐bonding complexation of their middle segments, the SAS/SES assembly forms a lamellar structure with alternating vitrified S and hydrogen‐bonded A/E association layers. The SAS/SES strip can be actuated and operate in response to environmental pH. The contraction ratio and working density of the SAS/SES actuator are approximately 50% and 90 kJ m^−3^, respectively; these values are higher than those of skeletal muscle fibers. In addition, the SAS/SES actuator shows a “catch‐state”, that is, it can maintain force without energy consumption, which is a feature of mollusc muscle but not skeletal muscle. This study provides a biomimetic approach for the development of artificial polymeric actuators with outstanding performance.

## Graphical abstract

A actuator with striated structure like skeletal muscle sarcomeres is fabricated by assembly of two triblock copolymers, polystyrene‐*b*‐poly(acrylic acid)‐*b*‐polystyrene (SAS) and polystyrene‐*b*‐poly(ethylene oxide)‐*b*‐polystyrene (SES). The actuator presents the “catch‐state” that maintains force while without energy consumption, and has high contraction ratio and working density compared with human skeletal muscle.

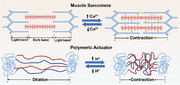

## 1 Introduction

Actuators are controllable work‐producing devices that use thermal, electrical, light, or chemical energy to achieve propulsion and manoeuvrability.^[^ ^1^, ^2^, ^3^, ^4^, ^5^, ^6^, ^7^, ^8^, ^9^, ^10^, ^11^, ^12^ ^]^ Steam engines, combustion engines, and electric motors are classical actuators that have driven the industrial revolution and shaped modern society.^[^ ^13^ ^]^ Different materials have been applied in the design of actuators, including stimulus‐responsive gels, liquid crystal elastomers, dielectric elastomers, shape memory polymers, conductive polymers, carbon nanotubes (CNTs), graphene, and polymer/inorganic nanocomposites.^[^ ^14^, ^15^, ^16^, ^17^ ^]^ Actuators obtained from different materials differ remarkably in their performance attributes, such as force, stroke, working density (*W*), power density (*P*), efficiency, life cycle, and frequency.^[^ ^3^ ^]^ Hunter et al. systematically explored artificial actuators produced from different types of materials and introduced their advantages, limitations, and applications.^[^ ^18^ ^]^

Actuation materials can be used in different forms, including bulk materials (3D), films (quasi 2D), and fibers (quasi 1D).^[^ ^19^, ^20^ ^]^ Actuating fibers are interesting materials because they are characterized by flexibility and anisotropy and could be further woven and knitted.^[^ ^21^, ^22^, ^23^ ^]^ Human movement is driven by the contraction and elongation of skeletal muscles, which have a fibrous structure.^[^ ^24^ ^]^ In the human body, skeletal muscles are attached to bones. Hundreds or thousands of muscle fibers bundled together make up an individual skeletal muscle. Each thin and long muscle fiber is surrounded by a sarcolemma (plasma membrane). The contractile units of the muscle fibers are myofibrils consisting of myofilaments.^[^ ^24^ ^]^ Myofilaments include thick myosin and thin actin filaments packed in an orderly manner. Skeletal muscle fibers exhibit a striated structure along the longitudinal direction, and the basic sliding unit of the myofibrils is called a sarcomere.^[^ ^25^ ^]^ Sarcomere contraction occurs when the thin filaments linked to the Z‐lines move closer along the thick filaments through the delicate interaction of myo‐ and tropomyosin proteins under a calcium ion gradient.^[^ ^26^, ^27^ ^]^

Inspired by human muscle, researchers have exerted considerable efforts to design and develop actuating fibers, especially hydrogel fibers, that show high similarity to human tissues.^[^ ^28^ ^]^ The hydrogel fibers of graphene oxide/alginate show actuation behavior through water adsorption,^[^ ^29^ ^]^ and hydrogel fibers coated with CNTs show actuation ability originating from the synergistic volume expansion of the hydrogel fibers and anisotropic restriction of aligned CNTs.^[^ ^30^ ^]^ Kim et al. reported a self‐helical hydrogel fiber that exhibited glucose‐responsive behavior; this fiber presented a maximum tensile stroke of 2.3% and maximum work density of 130 kJ m^−3^.^[^ ^31^ ^]^

Most artificial actuating fibers mimic the fibrous features of muscle fibers but not their delicate hierarchical structure. Skeletal muscle fibers feature striated structures characterized by closely packed thin and thick filaments.^[^ ^24^, ^25^ ^]^ This striated structure has served as an inspiration for attempts to construct imitation muscle.^[^ ^32^, ^33^, ^34^ ^]^ The alternate stacking of nondilation and dilation layers could lead to actuating hydrogel fibers, but this procedure is very time‐consuming.^[^ ^32^ ^]^ Electrostatic or magnetic fields could be used to fabricate layered structures in hydrogel systems, and actuation is realized by adjusting the temperature to induce phase transition and electrostatic permittivity variation.^[^ ^33^, ^34^ ^]^ The microphase separation of block copolymers can generate alternating lamellar domains that are similar to the striated structure of muscle fibers.^[^ ^35^, ^36^, ^37^ ^]^ If one domain remains fixed while the other dilates when exposed to certain stimuli, actuation behavior similar to that of skeletal muscles may be realized.

**Scheme 1:**
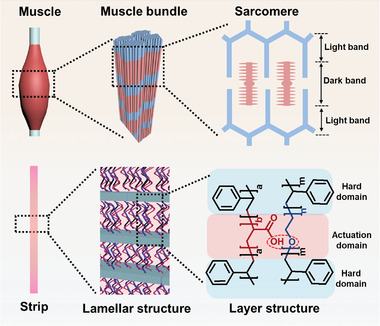
Striated structure of skeletal muscle and the lamellar structure of the polystyrene‐*b*‐poly(acrylic acid)‐*b*‐polystyrene (SAS)/polystyrene‐*b*‐poly(ethylene oxide)‐*b*‐polystyrene (SES) assembly.

To test this hypothesis, we used two triblock copolymers, polystyrene‐*b*‐poly(acrylic acid)‐*b*‐polystyrene (SAS) and polystyrene‐*b*‐poly(ethylene oxide)‐*b*‐polystyrene (SES), to mimic the striated structure of muscle fibers for actuation (**Scheme** **1**). Both SAS and SES have symmetrical structures featuring two outer polystyrene (S) blocks and inner blocks of poly(acrylic acid) (A) and poly(ethylene oxide) (E), respectively. The S block is incompatible with the A and E blocks, whereas the A and E blocks can form hydrogen‐bonded complexes.^[^ ^38^, ^39^, ^40^, ^41^, ^42^ ^]^ When SAS and SES are assembled, a lamellar structure with alternating vitrified S and hydrogen‐bonded A/E association layers is produced owing to the microphase separation of the triblock copolymers and the hydrogen‐bonding complexation of the A and E segments. In general, the hydrogen‐bonded complex of A/E homopolymers is sensitive to the environmental pH. As the pH increases, the complex dissociates and dissolves.^[^ ^40^, ^41^, ^42^ ^]^ However, the A and E blocks in the SAS/SES assembly are covalently linked to S blocks; hence, they are anchored to the vitrified S domain layers. In this case, the hydrogen bonds break and the A/E layers dilate but do not dissolve as the pH increases. When the pH decreases, the hydrogen bonds are rebuilt and the A/E layers contract. Therefore, the SAS/SES assembly can be used for actuation by adjusting the environmental pH.

## 2 Results and Discussion

**Figure 1:**
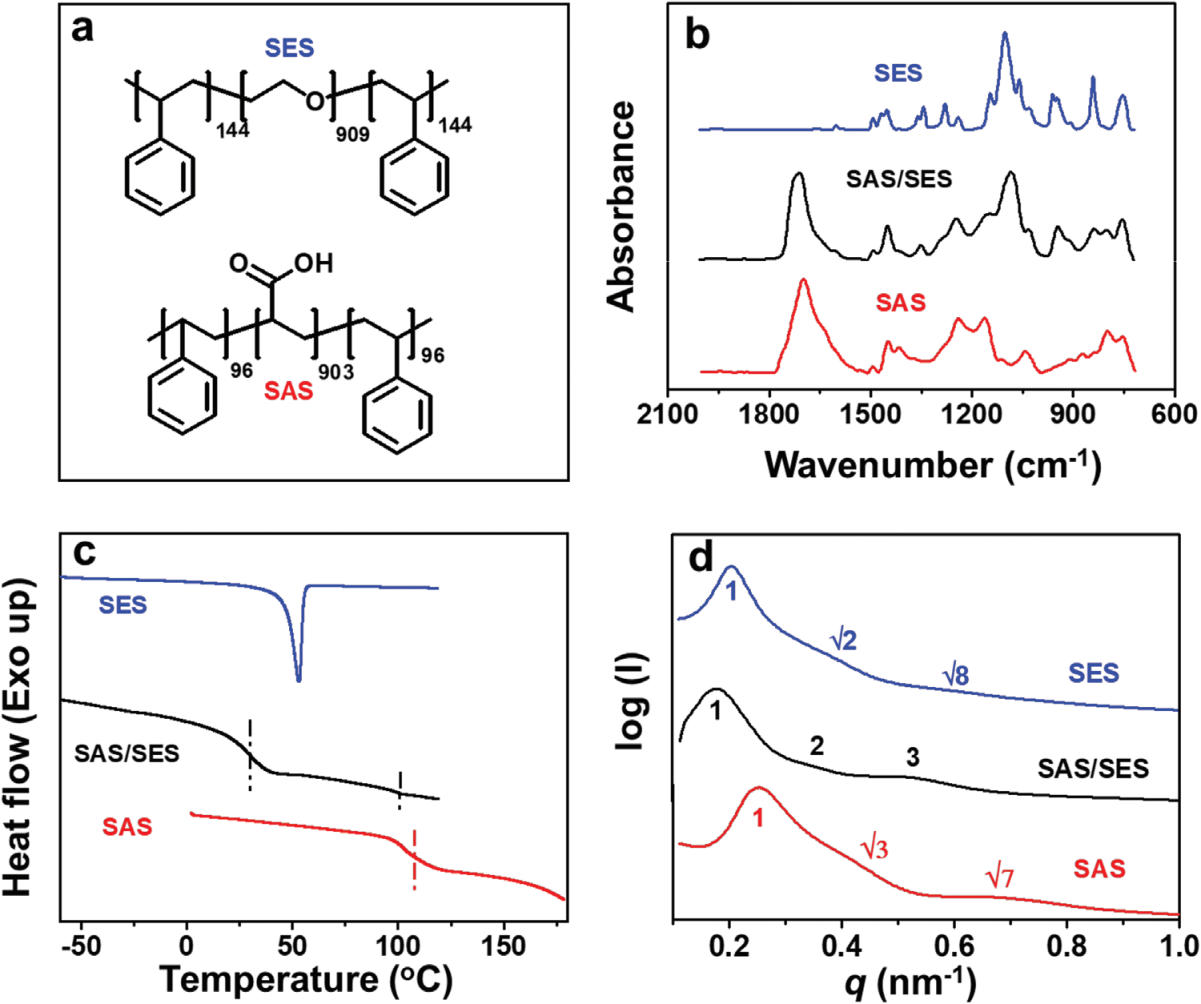
a) Chemical structures of the polystyrene‐*b*‐poly(ethylene oxide)‐*b*‐polystyrene (SES) and polystyrene‐*b*‐poly(acrylic acid)‐*b*‐polystyrene (SAS) triblock copolymers. b) Fourier transform infrared (FT‐IR) spectra, c) differential scanning calorimetry (DSC) curves, and d) small‐angle X‐ray scattering (SAXS) profiles of SES (blue lines), SAS (red lines), and the SAS/SES complex (black lines).

SAS and SES were synthesized via reversible addition–fragmentation chain transfer polymerization (Schemes S1 and S2 and Figures S1‐S6, Supporting Information). The average polymerization degrees of the S and A blocks of SAS are 96 and 903, respectively, while the average polymerization degrees of the S and E blocks of SES are 144 and 909, respectively (**Figure** **1**a). SAS and SES were dissolved and mixed in anhydrous *N*,*N*‐dimethylformamide (DMF) and subsequently cast into thin strips by solvent removal.

The Fourier transform infrared (FT‐IR) spectra shown in Figure 1b reveal a peak at 1697 cm^−1^, which could be attributed to the C=O stretching vibrations of COOH groups in SAS. The appearance of this peak at a wavenumber that is lower than that of free COOH (approximately 1740 cm^−1^) is caused by hydrogen‐bonding among the COOH groups of the A segments.^[^ ^43^ ^]^ In the FT‐IR spectrum of the SAS/SES complex, the peak located at 1725 cm^−1^ is attributed to C=O groups arising from the formation of hydrogen bonds between the ether and carboxylic acid groups.^[^ ^41^, ^42^ ^]^ The stretching vibrations of C–O–C in pure SES is observed at 1102 cm^−1^. Hydrogen‐bonding complexation causes this peak to move to 1083 cm^−1^.^[^ ^41^, ^42^, ^43^, ^44^ ^]^

The differential scanning calorimetry (DSC) curves of SAS, SES, and SAS/SES are shown in Figure 1c. The endothermal peak at 53 °C observed in the DSC curve of SES is assigned to the melting of E segment crystals. The S block domain of SAS exhibits a glass transition temperature at 107 °C. The DSC curve of the SAS/SES complex reveals the disappearance of the melting peak of E block crystals. Two glass transition temperatures are observed at 29 °C and 100 °C. The former is considered the glass transition temperature of hydrogen‐bonded A/E complex, which lies between the glass transition temperatures of the E (−60 °C) and A (106 °C) homopolymers, while the latter is the glass transition temperature of S block domains.^[^ ^41^, ^45^, ^46^ ^]^

According to the small‐angle X‐ray scattering (SAXS) data of SAS shown in Figure 1d, the scattered peaks of *q*_1_:*q*_2_:*q*_3_ = 1:√3:√7 indicate the formation of a hexagonally packed cylindrical phase. The three peaks of SES present a *q*_1_:*q*_2_:*q*_3_ ratio of 1:√2:√8, which roughly indicates a spherical phase.^[^ ^36^, ^38^ ^]^ When the two triblock copolymers are combined, the resultant SAS/SES complex exhibits a series of peaks of *q*_1_:*q*_2_:*q*_3_ = 1:2:3, which is characteristic of a lamellar structure.^[^ ^37^, ^47^ ^]^ Bright‐field transmission electron microscopy (TEM) images of thinly sectioned SAS/SES complex samples also confirm this lamellar structure (Figure S7, Supporting Information), with alternating S domain and A/E association layers.

**Figure 2:**
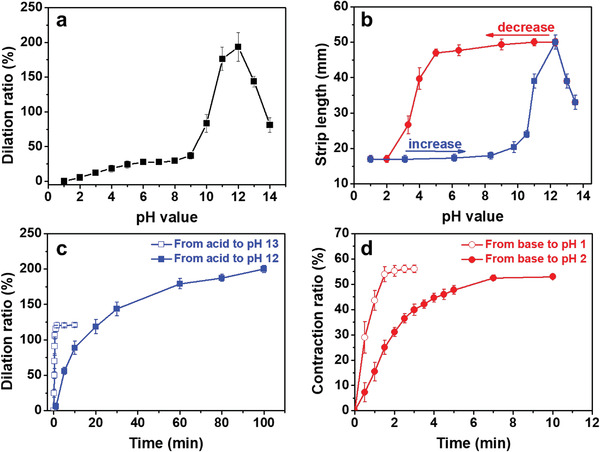
a) Dilation ratio of a polystyrene‐*b*‐poly(acrylic acid)‐*b*‐polystyrene (SAS)/polystyrene‐*b*‐poly(ethylene oxide)‐*b*‐polystyrene (SES) complex strip in solutions of different pH. b) Changes in strip length as a function of increasing environmental pH from 1 to 14 (red line) and decreasing pH from 14 to 1 (blue line). c) Changes in the dilation ratio of an SAS/SES strip over time after transfer from an acidic solution (pH = 1) to a basic solution (pH = 12 or 13). d) Changes in the contraction ratio of an SAS/SES strip over time after transfer from a basic solution (pH = 13) to an acidic solution (pH = 1 or 2). The original length and cross‐sectional area of the strip are 16 mm and 0.15 mm^2^, respectively. Each data point was obtained from at least five tests.

A series of SAS/SES strips of the same length was separately immersed in solutions of different pH. The dilation ratio (*ε*_d_; Equation 1) at equilibrium is plotted as a function of the solution pH (**Figure** **2**a). When the solution pH is lower than 8, the strip shows only slight dilation. At pH higher than 8, abrupt elongation is observed, and the strip stretches to its maximum length at pH = 12. Further increases in pH result in decreases in strip length because of the increase in ionic strength.^[^ ^48^, ^49^ ^]^ To illustrate this effect, we immersed the strips in a series of solutions at pH = 12 but with different concentrations of sodium chloride to achieve different ionic strengths. As the salt concentration increases, the strip length indeed shrinks (Figure S8, Supporting Information).

The FT‐IR spectra of SAS/SES strips incubated in solutions of different pH are shown in Figure S9 (Supporting Information). When the pH is higher than 8, the asymmetric stretching vibration of COO^−^ groups at 1550 cm^−1^ is observed, indicating the ionization of COOH groups in the A blocks. In this case, a complex composed of A and E homopolymers would dissolve because of the break‐down of hydrogen bonds.^[^ ^50^ ^]^ Because the A and E blocks are covalently linked to S blocks in the SAS/SES complex, they are anchored to the insoluble and vitrified S layers. Thus, at pH 8 or higher, the A/E complex domains swell, and the chains adopt a more expanded conformation when the interchain hydrogen bonds break, resulting in the dilation of the entire strip. The SAXS data of strip samples immersed in solutions of different pH confirm the expansion of the A/E domains (Figure S10, Supporting Information). The overall dilation of the SAS/SES strip could be attributed to the combined swelling of individual A/E layers.

The length of the SAS/SES strip is monitored over repeated increases and decreases in pH, as shown in Figure 2b. First, the strip was immersed in a solution of pH = 1. Next, the solution pH is gradually increased to 14 and then decreased back to 1. As the solution pH increases, the change in strip length shows the same trend observed in Figure 2a. When the pH decreases from 14 to 1, the strip length does not exhibit a distinct decrease until pH = 4. Changes in the strip length differ in the pH 3–11 region when the pH is cyclically increased and decreased (Figure 2b). However, at a low pH of 1–2 and high pH of 12–13, the strip shows nearly the same length, which is independent of the pH change (increasing or decreasing). Thus, if the strip is to be used for actuation, contraction should be conducted at pH lower than 3 and dilation should be performed at pH values higher than 11, where no discrepancy in actuator length is observed during cyclic increases and decreases in pH.

The dilation and contraction rates of the SAS/SES strip at pH = 12–13 and at pH = 1–2, respectively, are further investigated. Changes in *ε*_d_ (Equation 1) and contraction ratio (*ε*_c_, Equation 2) as functions of time are shown in Figure 2c,d, respectively. The dilation speed of the SAS/SES strip is at least 10× faster at pH = 13 than at pH = 12 (Figure 2c and Video S1, Supporting Information). Although the strip shows a higher dilation degree at pH = 12 than at pH = 13, considering the remarkable difference in dilation speed, pH = 13 was selected as the dilation working condition for further experiments. The equilibrium contraction length remains virtually unchanged at pH = 1 and 2 (Figure 2b), but the contraction speed is much faster at pH = 1 than at pH = 2 (Figure 2d and Video S2, Supporting Information). Therefore, pH = 1 was selected as the contraction working condition for further experiments.

**Figure 3:**
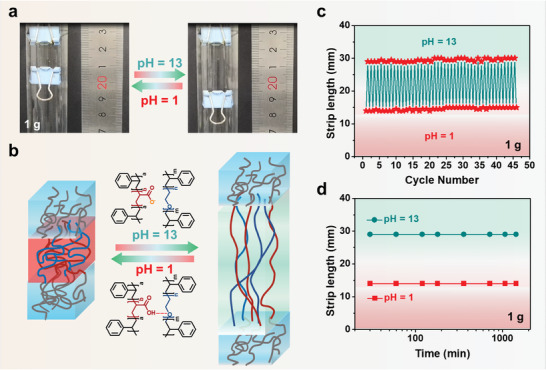
a) Actuation of a polystyrene‐*b*‐poly(acrylic acid)‐*b*‐polystyrene (SAS)/polystyrene‐*b*‐poly(ethylene oxide)‐*b*‐polystyrene (SES) strip loaded with cargo (dovetail clip, 1.0 g; gravity, 9.8 mN; and buoyancy, 1.3 mN) in solutions of pH = 1 and 13. b) Domain structures in the contraction (pH = 1) and dilation (pH = 13) states. c) Dilation–contraction cycles of the strip. d) Changes in the length of the strip loaded with a 1.0 g cargo as a function of time after contraction and dilation. The original length and cross‐sectional area of the strip are 16 mm and 0. 15 mm^2^, respectively.

When loaded with a cargo (dovetail clip, 1.0 g; gravity, 9.8 mN; and buoyancy, 1.3 mN), the SAS/SES strip quickly moves the cargo downward when the solution pH is adjusted to 13 and lifts the cargo upward when the pH is adjusted to 1 (**Figure** **3**a and Videos S3 and S4, Supporting Information). The density of the strip (*ρ*_s_) in the dry state is *ρ*_s_ = 1.1 g cm^−1^, and the strips sink to the bottom in solutions of pH 1 and 13 (Figure S11, Supporting Information). In this experiment, the weight of the cargo is 200 times that of the dry weight of the strip. Work is produced by the contraction of the strip.

At pH = 13, the hydrogen bonds between the A and E blocks break, causing the swelling of the A/E domain and dilation; when the pH value is adjusted to 1, the carboxylate groups are protonated, and hydrogen bonds re‐form; thus, the A/E domain contracts to lift the cargo upward (Figure 3b). When the environmental pH is alternately adjusted between 1 and 13 repeatedly, the cargo‐loaded strip shows reliable contraction and dilation (Figure 3c), with a 1–2 s latency and complete actuation in 20 s (Figure S12, Supporting Information).

To validate the role of the vitrified S domains, we assembled the triblock polymers SES and SAS with A and E homopolymers, respectively, and then tested the resulting A/SES and SAS/E strips for actuation under the same conditions. The A/SES strip is stable in acidic solution but broken at pH = 13 (Video S5, Supporting Information). The SAS/E strip quickly dilates as the pH increases from 1 to 13 but does not contract when the pH is returned to 1 (Video S6, Supporting Information). If the A or E segment chains are not anchored to the vitrified S‐domain layer, they dissolve at pH 13. The IR spectra demonstrate that the A homopolymers are dissolved from the A/SES strip while the E homopolymer is dissolved from the SAS/E strip (Figure S13, Supporting Information). These results prove that the anchoring of the A and E chains to the insoluble and vitrified S domains is essential to achieve successful actuation.

The length of the SAS/SES strip is nearly fixed when contraction at pH = 1 or dilation at pH = 13 is completed (Figure 3d), which means the strip does not creep when loaded with cargo in either the contraction or dilation state. The SAS/SES strip can maintain force in the contraction/dilation state without energy consumption. In nature, this behavior is characteristic of mollusc muscle and is referred as the “catch‐state".^[^ ^51^, ^52^ ^]^ By contrast, the skeletal muscles of mammals require constant energy inputs to maintain force and, thus, do not show the "catch‐state” behavior.

**Figure 4:**
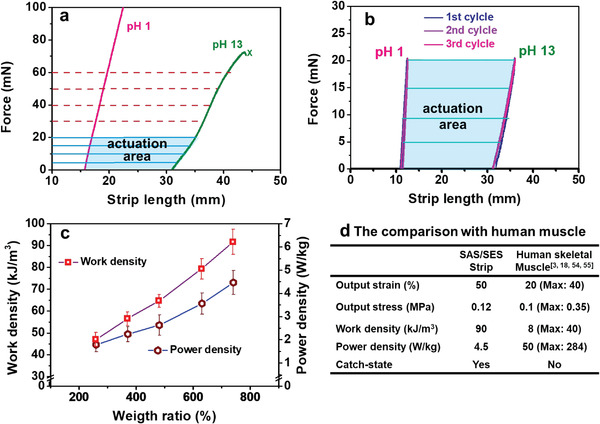
a) Force–length curves of the polystyrene‐*b*‐poly(acrylic acid)‐*b*‐polystyrene (SAS)/polystyrene‐*b*‐poly(ethylene oxide)‐*b*‐polystyrene (SES) strip at the equilibrium contraction (pH = 1) and dilation (pH = 13) states. b) Linear regions of the loading/unloading curves of the SAS/SES strip over three consecutive cycles. c) Plots of the working and power densities of the SAS/SES strip as a function of the weight ratio of the cargo and strip. d) Comparison of the SAS/SES artificial actuator and human skeletal muscle. Each data point is obtained from at least five tests. The original length and cross‐sectional area of the strip are 16 mm and 0. 15 mm^2^, respectively.

To investigate the working capacity of the SAS/SES strip further, we determined its mechanical properties in the contraction (pH = 1) and dilation (pH = 13) states. Changes in the force applied to the strip as a function of the strip length are shown in **Figure** **4**a. The force versus strip length plots in both states show a linear region. In this region, the loading and unloading curves in both contraction and dilation states nearly overlap (Figure 4b), which indicates ideal elastic behavior. Beyond this region, hysteresis could be observed (Figure S14, Supporting Information). The SAXS results illustrate irrecoverable structural breakage when the strain exceeds well beyond the linear region (Figure S15, Supporting Information). Considering the hysteresis and structural breakage observed, the actuation of the SAS/SES strip should be conducted in its linear region to produce reliable and durable performance.

The *W* and *P* of the SAS/SES actuator system are determined at different load weights in the linear mechanical region. The weight ratio (*α*) is calculated as cargo weight divided by the dry weight of the strip (Equation 3). The work done by the strip to lift cargo can be calculated using the force produced (Equation 4) and movement distance, which are obtained from experimental measurements. *W* (Equation 5, J m^−3^) is then calculated by normalizing the work by the volume of the strip, while the energy density (*E*, Equation 6, J kg^−1^) is obtained by normalizing the output work by the mass of the strip. *P* (Equation 7, W kg^−1^) is calculated by dividing *E* by the actuation time. The actuation force linearly increases with increasing cargo weight, as demonstrated by Equation 4. The actuation time does not change significantly as the cargo weight increases. When the pH values for dilation and contraction are fixed, the actuation time is controlled by the diffusion process, as determined from the cross‐sectional area of the strip.^[^ ^53^ ^]^ As the cargo weight changes, the cross‐sectional area shows very small variations. Plots of *W* and *P* as functions of *α* are shown in Figure 4c; here, *W* and *P* similarly increase as *α* increases.

The SAS/SES strip and human skeletal muscle are compared in Figure 4d. The output strain (represented by *ε*_c_) of the SAS/SES strip is approximately 50% (Figure S16, Supporting Information), which is higher than that of mammalian skeletal muscles. The normal output strain of skeletal muscle is approximately 20% owing to joint restriction.^[^ ^18^, ^54^ ^]^ The output stress of the SAS/SES strip is 0.12 MPa, which is close to that of mammalian skeletal muscles (0.1 MPa, max. 0.35 MPa).^[^ ^18^, ^54^ ^]^ The *W* of the SAS/SES strip can reach 90 kJ m^−3^, which is much higher than that of human skeletal muscles (8 kJ m^−3^, max. 40 kJ m^−3^).^[^ ^3^, ^18^, ^54^ ^]^ However, its *P* (4.5 W kg^−1^) remains lower than that of human skeletal muscles.^[^ ^18^ ^]^ pH‐driven actuation is a diffusion‐controlled process. As the cross‐section of the strip area decreases, the diffusion time is reduced ($\tau \;=\frac{A}{2D}\;$, where *τ* is the diffusion time, *A* is the cross‐sectional area of the strip, and *D* is the diffusion coefficient); hence, the response time and *P* increase.^[^ ^55^ ^]^ When the strip becomes thinner, its *P* may approach or even exceed that of the skeletal muscle.

A theoretical method can be used to calculate the energy efficiency from chemical energy to mechanical work of this type of pH‐driven hydrogel actuator.^[^ ^56^ ^]^ The chemical energy is deduced from the change in the chemical potential of the pH increasing/decreasing cycle, and the mechanical work is determined by direct measurement. During this calculation, the energy that pumps H^+^ and OH^−^ is ignored, although it is important for practical operation. Osada estimated that the energy efficiency of a pH‐driven actuator is approximately 40%, which is higher than that of traditional gasoline engines (20–30%) and lower than that of skeletal muscle (50%).^[^ ^57^ ^]^ The development of an artificial actuators that can achieve the energy transfer efficiency of skeletal muscle both highly desirable and a great challenge.^[^ ^3^, ^18^ ^]^

## 3 Conclusion

In summary, a pH‐driven artificial actuator was fabricated by assembling two triblock copolymers, namely, SAS and SES. The SAS/SES actuator has a lamellar structure inspired by the striated structure of skeletal muscle sarcomeres. Microphase separation and hydrogen‐bonding endowed the SAS/SES assembly with a lamellar structure featuring alternating vitrified S‐domain and pH‐sensitive A/E association layers. As the pH increases–decreases, the A/E association layers show reversible dilation–contraction, whereas the S domain layers remain stable. In addition, the A and E blocks are covalently anchored to the vitrified S domain layers and do not dissolve as the pH increases; hence, reliable and durable actuation is produced. The SAS/SES strip demonstrates excellent actuator performance, yielding a higher output strain and working density than those produced by human skeletal muscles. Moreover, the SAS/SES actuator can maintain force without energy consumption after actuation, similar to the “catch‐state” of mollusc muscle. This study provides a biomimetic approach for the development of artificial polymeric actuators with outstanding performance.

## 4 Experimental Section

### Materials

The synthetic details of the SAS and SES are described in the Supporting Information. DMF (99%, Greagent) was dried over MgSO_4_ for 48 h at room temperature and distilled through a high‐vacuum line into a solvent storage bottle before use. HCl (AR, 36%, Greagent) and NaOH (AR, 96%, Greagent) were used as received.

### Preparation of the SAS/SES Actuator

SAS and SES were separately dissolved in anhydrous DMF at concentrations of 5wt%. The two solutions were mixed at 50 °C with stirring for 12 h and then poured into a Teflon mold (30 × 20 × 0.5 mm). The DMF was removed by drying in a vacuum oven at 50 °C for 48 h. The film was cut into strips measuring 20 × 4 × 0.038 mm in size. Prior to testing, the strips were dried thoroughly in a vacuum oven.

### Characterization

FT‐IR spectra were recorded on a Bruker spectrometer (Vertex 70) with a single‐bounce attenuated total reflection attachment. DSC analysis was performed on a TA Discovery DSC 250 instrument at a heating rate of 10 °C min^−1^ under a nitrogen atmosphere flowing at a rate of 50 mL min^−1^. The SAXS data were collected at beamline BL16B1 of the Shanghai Synchrotron Radiation Facility. The distance between the sample and detector was 1800 mm, and the X‐ray wavelength was 0.124 nm. TEM measurements were conducted using a JEM‐2100 transmission microscope (JEOL, Japan) with an accelerating voltage of 200 kV. The strip was embedded in epoxy resin and cut into slices measuring 100–200 nm thickness using an ultrathin frozen slicer (Leica EM UC7). The ultrathin sections were ultrasonically dispersed in ethanol and fished onto a copper grid. After drying, sections were stained with ruthenium tetroxide. Mechanical tests were performed using a Suns UTM2502 universal testing machine with a stretching speed of 10 mm min^−1^.

### Metrics Definition and Calculation

Dilation ratio (*ε*_d_): *ε*_d_ is calculated as the difference between the strip length in the dilation state (*l*_d_) and the strip length in the contraction state (*l*_c_) divided by the strip length in the contraction state (*l*_c_).

(1) $$\varepsilon_{d}=\left(l_{d}-l_{c}\right)/l_{c}$$

Contraction ratio (*ε_c_*, output strain): The contraction ratio is the length change of the contraction normalized by the length at the dilation state (*l*_d_).

(2) $$\varepsilon_{c}=\left(l_{d}-l_{c}\right)/l_{d}$$

Weight ratio (*α*): *α* is the ratio of the cargo weight (*m*_c_) to the dry weight of the SAS/SES strip (*m*_s_).

(3) $$\alpha =m_{c}/m_{s}$$

Output stress (*σ*): *σ* is the generated force normalized by the initial cross‐sectional area (*A*) of the strip actuator.

(4) $$\sigma =F/A=m_{c}\left(1-\rho_{w}/\rho_{c}\right)g/A$$

where *g* is the gravity constant, *ρ*_c_ is the cargo density, and *ρ*_w_ is the water density.

Working density (*W*): *W* is the output work generated by the SAS/SES strip normalized by the volume of the strip (*V*_s_).

(5) $$W=\left(l_{d}-l_{c}\right)m_{c}\left(1-\rho_{w}/\rho_{c}\right)g/V_{s}$$

Energy density (*E*): *E* is the output work generated by the SAS/SES strip normalized by the dry mass of the strip (*m*_s_).

(6) $$E=\left(l_{d}-l_{c}\right)m_{c}\left(1-\rho_{w}/\rho_{c}\right)g/ms$$

Power density (*P*): *P* is the *E* normalized by the actuation time of strip (*t*).

(7) P=E/t

The density and mass fraction of polystyrene are *ρ*_St_ = 1.05 g cm^−3^ and 32%, respectively; those of poly(ethylene oxide) are *ρ*_E_ = 1.20 g cm^−3^ and 26%, respectively, and those of poly(acrylic acid) are *ρ*_A_ = 1.09 g cm^−3^ and 42%, respectively. The density of the strip was calculated as *ρ*_s_ = 1.1 g cm^−3^.

### Statistical Analysis

The data were processed and calculated according to the procedures described in Section Metrics Definition and Calculation. All data were presented as mean ± standard deviation. All the samples used for the tests measured 16 × 4 × 0.038 mm. One‐way analysis of variance was used to conduct statistical analysis, and statistical significance was considered at *p* < 0.05. Statistical analysis was performed using the Origin 8.5 software.

## Conflict of Interest

The authors declare no conflict of interest.

## Supporting information

Supporting Information

Supplemental Video 1

Supplemental Video 2

Supplemental Video 3

Supplemental Video 4

Supplemental Video 5

Supplemental Video 6

## Data Availability

Research data are not shared.
