# Supplementary material for: Skeletal Muscle Fibers Inspired Polymeric Actuator by Assembly of Triblock Polymers
Source: Adv Sci (Weinh). 2022 Mar 6;9(13):2105764. doi: 10.1002/advs.202105764 (PMC9069194; doi:10.1002/advs.202105764)
Supplement: Supplementary file 1 — Supporting Information [file ADVS-9-2105764-s007.pdf]

ADVANCED SCIENCE
Open Access

Supporting Information

for *Adv. Sci.*, DOI 10.1002/advs.202105764

Skeletal Muscle Fibers Inspired Polymeric Actuator by Assembly of Triblock Polymers

*Weijie Wang, Xian Xu, Caihong Zhang, Hao Huang, Liping Zhu, Kan Yue, Meifang Zhu and Shuguang Yang**

Supporting Information

for

Skeletal Muscle Fibers Inspired Polymeric Actuator by Assembly of Triblock Polymers

Weijie Wang[1], *Xian Xu*[1], *Caihong Zhang*[1], *Hao Huang*[1], *Liping Zhu*[1], *Kan Yue*[2], *Meifang Zhu*[1], *Shuguang Yang*[1 *]

[1] State Key Laboratory for Modification of Chemical Fibers and Polymer Materials, Center for Advanced Low-dimension Materials, College of Materials Science and Engineering, Donghua University, Shanghai 201620, P. R. China

[2] South China Advanced Institute for Soft Mater Science and Technology, School of Molecular Science and Engineering, South China University of Technology, Guangzhou 510640, P. R. China

*To whom correspondence should be addressed: shgyang@dhu.edu.cn

Table of Contents

1. Synthesis

Scheme S1 Synthesis route for the SES triblock copolymer.

Scheme S2 Synthesis route for the SAS triblock copolymer.

2. Supporting Figures

Figure S1 ^{1}H NMR spectra of (a) RAFT-yne, (b) RAFT-PEO-RAFT and (c) SES.

Figure S2 FT-IR spectra of RAFT-PEO-RAFT and N_3-PEO-N_3.

Figure S3 GPC curves of homopolymer PEO and target product of triblock copolymer SES.

Figure S4 GPC curves of homopolymer PS and intermediate product of triblock copolymer PS-PtBA-PS.

Figure S5 FT-IR spectra of PS-PtBA-PS and the target triblock copolymer SAS.

Figure S6 ^{1}H NMR spectra of SAS showing that the deprotection reaction was completed.

Figure S7 TEM image of thin-sectioned SAS/SES strip sample showing the microphase separated lamellar structure.

Figure S8 Lengths of the SAS/SES strips immersed in solutions of pH = 12 but different NaCl concentrations.

Figure S9 FT-IR spectra of the SAS/SES strips equilibrated in the solutions of different pH values.

Figure S10 SAXS profiles of the SAS/SES strips equilibrated in the solutions of different pH values.

Figure S11 Photos to illustrate the strip will not produce buoyancy and affect its driving performance.

Figure S12 Actuation of the SAS/SES strip with a cargo of 1.0 g when alternately placed in pH = 1 and pH = 13 solutions.

Figure S13 FT-IR spectra of the SAS/SES, SAS/E and A/SES strips after treatment by pH = 13 solution.

Figure S14 One cyclic stress-strain curve of the SAS/SES strip at pH = 1 showing the hysteresis beyond linear region.

Figure S15 2D SAXS pattern of SAS/SES before and after stretch heavily (beyond linear region).

Figure S16 Output strain of the SAS/SES strips determined at different weight ratio.

4. Supporting Videos

Video S1 The dilation of the SAS/SES strip as pH increasing.

Video S2 The contraction of the SAS/SES strip as pH decreasing.

Video S3 The SAS/SES strip alternately dipping into pH = 1 solution and pH = 13 solution.

Video S4 The contraction and dilation of SAS/SES by adding acid and base into the solution.

Video S5 The breakage of A/SES strip as pH increasing to pH = 13.

Video S6 The elongation of SAS/E strip as pH increasing to pH = 13 but no contraction as pH decreasing to pH = 1.

1. Synthesis

Materials. N_3-PEO_{909}-N_3 (M_n = 40 kg mol^{-1}, PDI = 1.01) was purchased from Sinopeg Chemical Reagent Co., 3-Butyn-1-ol (98%, Adamas), 4-dimethylaminopyridine (DMAP, 98%, Adamas), *N,N′*-diisopropylcarbodiimide (DIPC, 98%, Adamas), *N,N,N′,N″,N″*-pentamethyl diethylenetriamine (PMDETA, 95%, Adamas), trifluoroacetic acid (TFA, 98%, Maklin), diethyl ether (99%, Greagent), tetrahydrofuran (THF, 99%, Greagent), 1,4-dioxane (99%, Greagent), petroleum ether (PE, 95%, b.p. 60-90 °C, Greagent), ethyl acetate (EA, 99.6%, Greagent) were used as received. Dichloromethane (DCM, CH_2Cl_2, 99%, Greagent) was purified by distillation from CaH_2. Toluene (99%, Greagent) was dried over CaH_2 for 24 h at room temperature and distilled through a high vacuum line into a solvent storage bottle containing 4 Å molecular sieves. CuBr (98%, Maklin) was purified by stirring in acetic acid for 72 h, filtering and washing with acetone, and stored under a nitrogen atmosphere. 2,2'-Azobis(2-methylpropionitrile) (AIBN, 98%, Macklin) was recrystallized three times in ethanol. Styrene (99%, Aladdin) and *tert*-butyl acrylate (tBA, 98%, Macklin) were purified by passing a basic alumina column to remove the inhibitor before use. *S*-1-Dodecyl-*S′*-(α,α'-dimethyl-α''-acetic acid)trithiocarbonate (RAFT-COOH) and *S,S′*-bis(α,α'-dimethyl-α''-acetic acid)-trithioccarbonate (HOOC-RAFT-COOH) were prepared according to the reported procedure.[1]

Characterization. ^{1}H nuclear magnetic resonance (NMR) spectra were acquired using $CDCl_3$ and d_6-DMSO as solvent (99% D, J&K Chemicals) with a Bruker 600 MHz NMR spectrometer. The ^{1}H NMR spectra were referenced to the residual proton impurities in $CDCl_3$ at δ 7.27 ppm and DMSO at δ 2.50 ppm. Gel permeation chromatography (GPC) was performed to using Waters Instrument (515/2410/2487) with THF as the eluent at a flow rate of 1.0 mL min^{-1} at 40 °C and polystyrenes as standards. Fourier transform infrared (FT-IR) spectra was recorded on a Bruker spectrometer (Vertex 70) with the Single-Bounce ATR attachment.

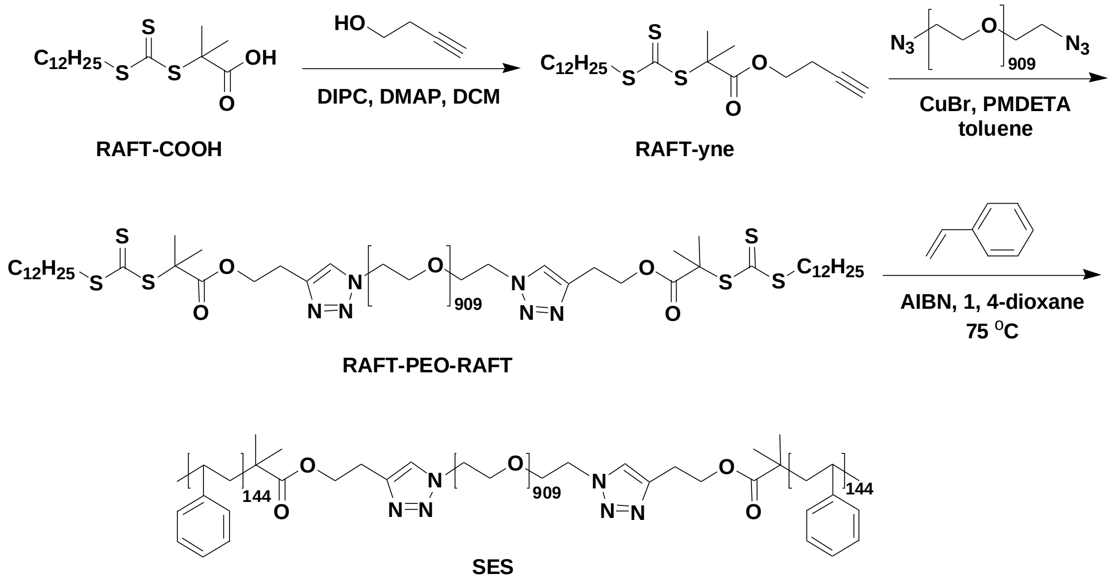

Scheme S1 Synthesis route for the SES triblock copolymer.

Synthesis of RAFT-yne. RAFT-COOH (20 g, 54.94 mmol), 3-butyn-1-ol (6.46 g, 66.00 mmol) and DMAP (0.34 g, 3.00 mmol) were dissolved in 40 mL anhydrous DCM, and the mixture was cooled to 0 ℃ in an ice-bath. DIPC (8.32 g, 66.00 mmol) was slowly added with a syringe. The reaction mixture was allowed to room temperature and stirred for overnight. When the reaction was finished, the crude production was purified by column chromatography on a silica gel column with a mixture of PE and EA as the eluent (10:1, v/v). The product RAFT-yne was collected as a yellow powder with a yield of 91%. ^{1}H NMR (600 MHz, $CDCl_3$, ppm, δ): 4.18-4.20 (t, 2H), 3.24-3.26 (t, 2H), 2.50-2.53 (t, 2H), 1.95-1.96 (s, 1H), 1.68 (s, 6H), 1.63-1.67 (m, 2H), 1.24-1.37 (m, 18H), 0.85-0.87 (t, 3H).

Synthesis of RAFT-PEO-RAFT. In a 100 mL Schlenk flask equipped with a magneton, N_3-PEO_{909}-N_3 (4 g, 0.1 mmol), RAFT-yne (6.66 g, 16 mmol) and CuBr (4 mg, 0.028 mmol) were added followed by the addition of 50 mL anhydrous toluene. The mixture was degassed three times by freeze-vacuum-thaw cycles. Then PMDETA (5.3 mg, 0.028 mmol) was added into the mixture under nitrogen atmosphere and degassed once again by the freeze-vacuum-thaw cycle. The reaction mixture was stirred at room temperature for 48 h. After that, the mixture was concentrated by rotary evaporator and purified by column chromatography on a silica gel column. The column was first eluted with DCM to recollect

the excess starting material RAFT-yne, and then further eluted with a mixture of chloroform and methanol (10:1, v/v) to get the product RAFT-PEO_{909}-RAFT. The crude product was precipitated into cold ether for further purification. After filtration, the purified product RAFT-PEO_{909}-RAFT was collected as a pale-yellow powder with a yield of 77%. ^{1}H NMR (600 MHz, $CDCl_3$, ppm, δ): 7.54 (s), 4.48-4.50 (t), 4.35-4.37 (t), 3.63 (s,), 3.04-3.07 (t), 1.66 (s).

Synthesis of SES. To a 50 mL flask equipped with a magneton were added RAFT-PEO_{909}-RAFT (2 g, 0.05 mmol), initiator AIBN (0.04 g, 0.02 mmol), styrene (10 mL, 87.0 mmol) and 10 mL anhydrous toluene as the solvent. The mixture was degassed with three freeze-pump-thaw cycles and placed into an oil bath at 75 °C for polymerization. After the polymerization was finished, the flask was quenched into liquid nitrogen. And the crude product was precipitated into cooled ether three times to thoroughly remove the unreacted styrene monomer. After filtration, the target triblock copolymer product SES was obtained as a white powder. $M_{n,NMR}$= 70 k. $M_{n,PS\ block}$ = 30 k. PDI_{GPC} = 1.09. ^{1}H NMR (600 MHz, $CDCl_3$, ppm, δ): 6.37-7.10 (m, 1440H), 3.63 (s, 3636H), 1.75-2.00 (m, 288H), 1.25-1.50 (m, 576H).

Discussions on the synthesis of SES. The synthetic procedure of SES triblock copolymer is shown in the **Scheme S1**. The chemical structure of RAFT-yne was proved by ^{1}H NMR (**Figure S1a**). The "click" reaction of RAFT-yne with N_3-PEO_{909}-N_3 afforded the macromolecular RAFT agent RAFT-PEO_{909}-RAFT, the structure of which was confirmed by FT-IR and ^{1}H NMR spectra. As shown in **Figure S2**, the vibrational peak of the azide group at 2111 cm^{-1} totally disappeared after the "click" reaction, confirming the successful reaction with RAFT-yne. Moreover, in the ^{1}H NMR spectra in **Figure S1b**, besides the strong resonance at 3.63 ppm from the methylene protons in the PEO main chain, the chemical shifts of methylene protons adjacent to the alkyne group moved from 2.51 ppm to 3.06 ppm, and the methylene protons close to the ester group shifted from 4.20 ppm to 4.35 ppm. In addition, the proton in the alkynyl group at 1.95 ppm fully disappeared, while a new weak resonance peak appeared at 7.54 ppm, which was attributed to the proton in the newly generated heterocyclic rings. Finally, the macromolecular RAFT agent was applied to polymerize styrene to prepare the target triblock copolymer SES. As shown in **Figure S1c**, a

set of resonances in the range of 6.44 and 7.08 ppm appeared, which can be attributed to the protons of benzene ring in the PS block. Combined with GPC curves (**Figure S3)**, the SES triblock copolymer showed a monodisperse peak, and its retention time was smaller than that of RAFT-PEO_{909}-RAFT, due to the increase in molecular weight. Based on these data, the successful preparation of the **SES** triblock copolymer was validated. The molecular weight of a PS segment determined by ^{1}H NMR was 15k, and average polymerization degree was 144. *PDI* of SES determined by GPC with PS standard sample was 1.09.

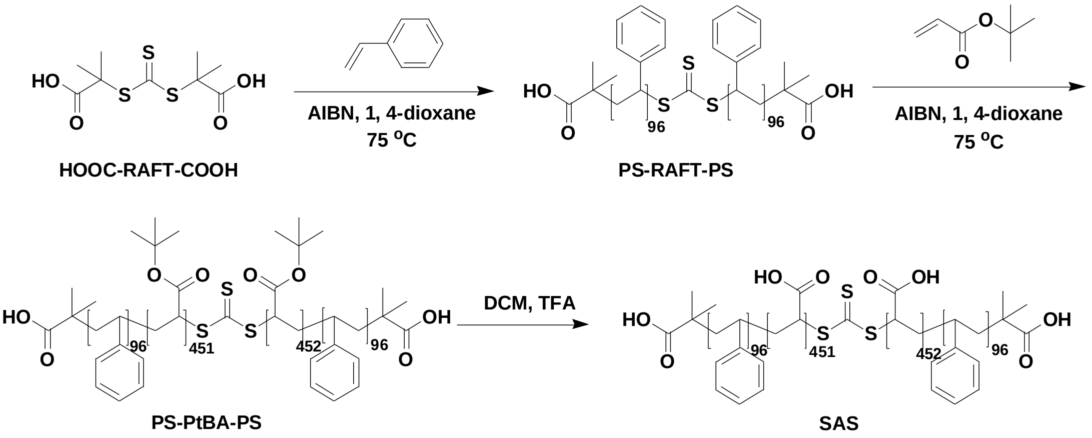

Scheme S2 Synthesis route for the SAS triblock copolymer.

Synthesis of PS-RAFT-PS. The SAS triblock copolymer was synthesized according to the previously reported procedure with modifications.[1, 2] First, the RAFT agent HOOC-RAFT-COOH (0.010 g, 0.035 mmol), initiator AIBN (0.020 g, 0.010 mmol) and 50 mL of styrene monomer were added into a 100 mL of Schlenk flask with a magneton. After degassed three times by freeze-vacuum-thaw cycles, the flask was placed in an oil bath at 75 °C for polymerization. The degree of polymerization was monitored by GPC. When reaching the desired PS molecular weight, the polymerization was stopped by quenching the mixture into liquid nitrogen. Then the mixture was precipitated into methyl alcohol three times to thoroughly remove the unreacted styrene monomer. After filtration, the target product of PS-RAFT-PS was collected. The molecular weight of a PS segment determined by GPC was 10k, average polymerization degree was 96, and *PDI* was 1.07.

Synthesis of PS-PtBA-PS. The macromolecular RAFT agent PS-RAFT-PS (2.0 g, 0.2 mmol),

initiator AIBN (0.04 g, 0.02 mmol), *tert*-butyl acrylate (30 mL, 0.21 mmol) and 1,4-dioxane (20 mL) as the solvent were added into a 100 mL Schlenk flask with a magneton. The mixture was degassed three times by freeze-vacuum-thaw cycles and placed it into a 75 °C oil bath for polymerization. The degree of the polymerization was monitored by ^{1}H NMR. When reaching the desired PtBA molecular weight, the flask was quenched into liquid nitrogen to stop the polymerization. The reaction mixture was then precipitated into a mixture solution of water/methanol (1:1, v/v) three times to afford the product PS-PtBA-PS. With the PS segments as the reference, the molecular weight of PtBA was 116k, and average polymerization degree was 903. GPC curves shown in **Figure S4** confirmed a monodispersed peak for PS-PtBA-PS, which shifted to a lower retention time relative to that of PS_{96}-RAFT-PS_{96} due to an increase in molecular weight.

Synthesis of SAS triblock copolymer. The target triblock copolymer SAS was obtained by hydrolysis of the PtBA block to remove the *tert*-butyl ester groups.[2] 3 g PS-PtBA-PS was dissolved in a mixture of 30 mL DCM and 30 mL TFA. The mixture was stirred at room temperature for 48 h and the hydrolysis reaction was monitored by FT-IR and ^{1}H NMR spectra. In the FT-IR data (**Figure S5**), the C=O absorbance peak moved from 1721 cm^{-1} to 1697 cm^{-1}, and the peak of *tert*-butyl groups at 1365 cm^{-1} thoroughly disappeared after the reaction. After the deprotection reaction, the peak of the *tert*-butyl groups at around 1.40 ppm fully disappeared (**Figure S6**). After the reaction finished. the solvent was removed by rotary evaporator, and the crude product was dissolved in THF and precipitated into cooled diethyl ether to obtain white powder of SAS.

2. Supporting Figures

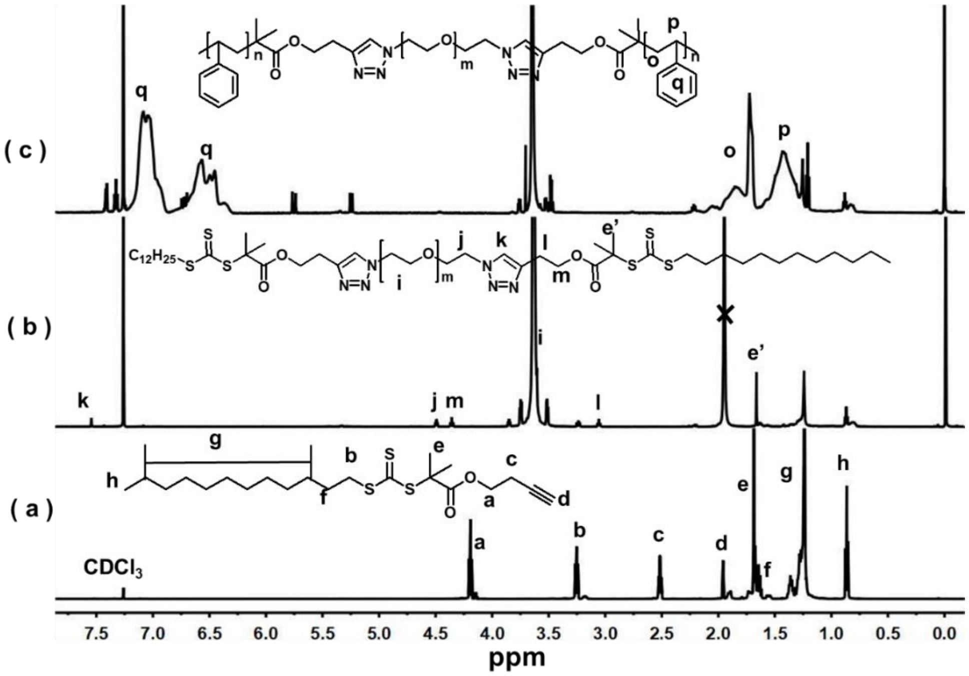

Figure S1 ^{1}H NMR spectra of (a) RAFT-yne, (b) RAFT-PEO-RAFT and (c) SES. Assignments of major peaks were marked in the spectra.

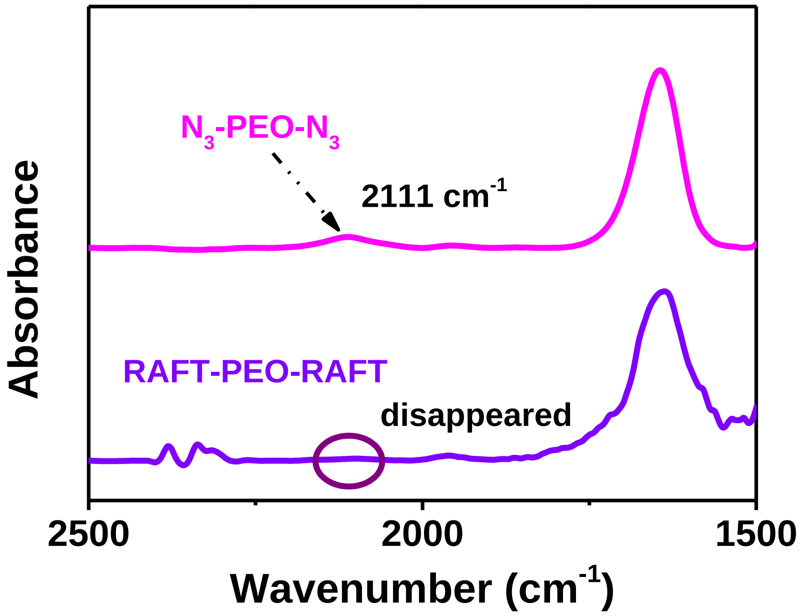

Figure S2 FT-IR spectra of RAFT-PEO-RAFT (purple line) and N_3-PEO-N_3 (pink line).

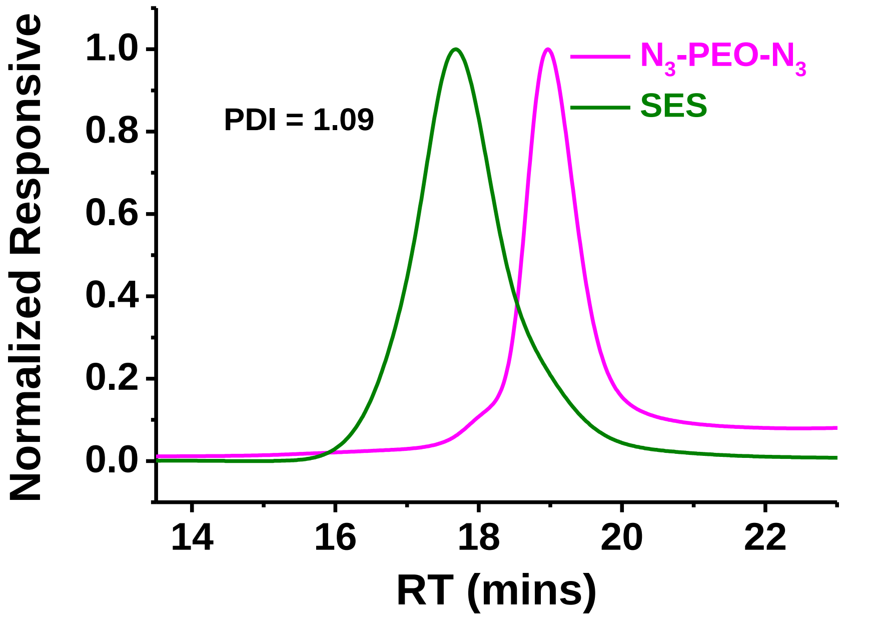

Figure S3 GPC curves of N_3-PEO-N_3 (pink line) and the target triblock copolymer SES (green line).

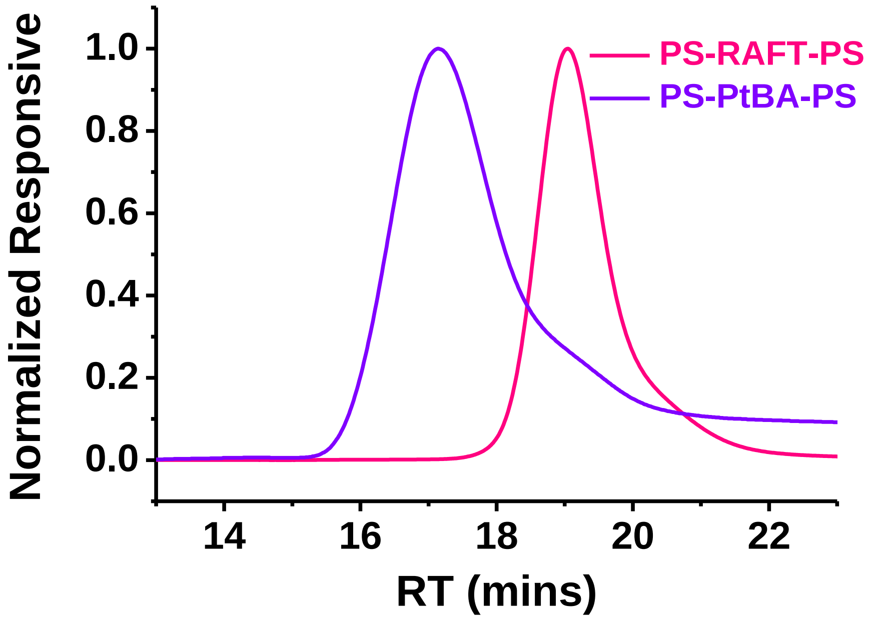

Figure S4 GPC curves of PS-RAFT-PS (pink line) and the intermediate product of triblock copolymer PS-PtBA-PS (purple line).

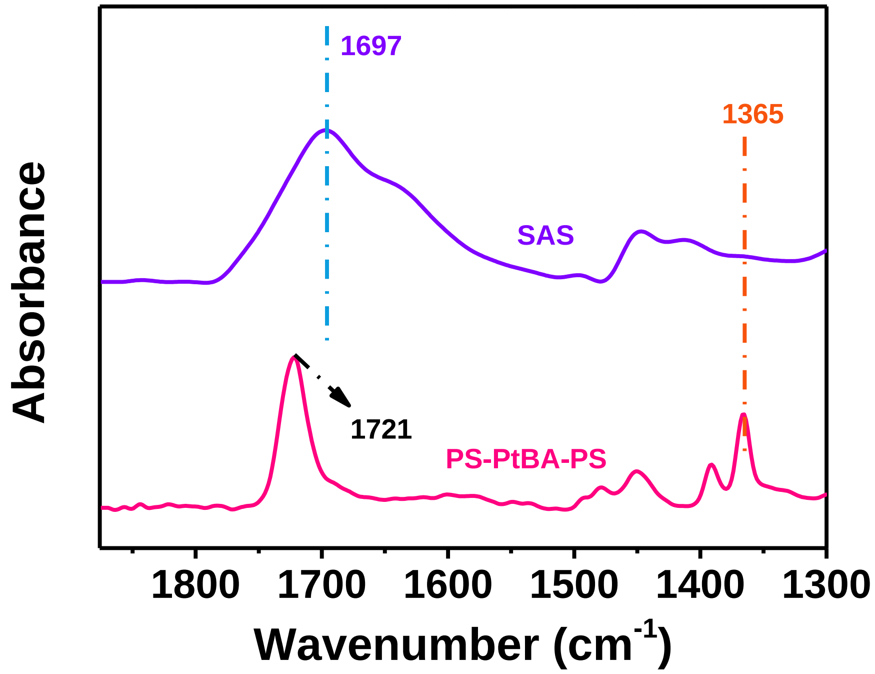

Figure S5 FT-IR spectra of PS-PtBA-PS (pink line) and the target triblock copolymer SAS (purple line).

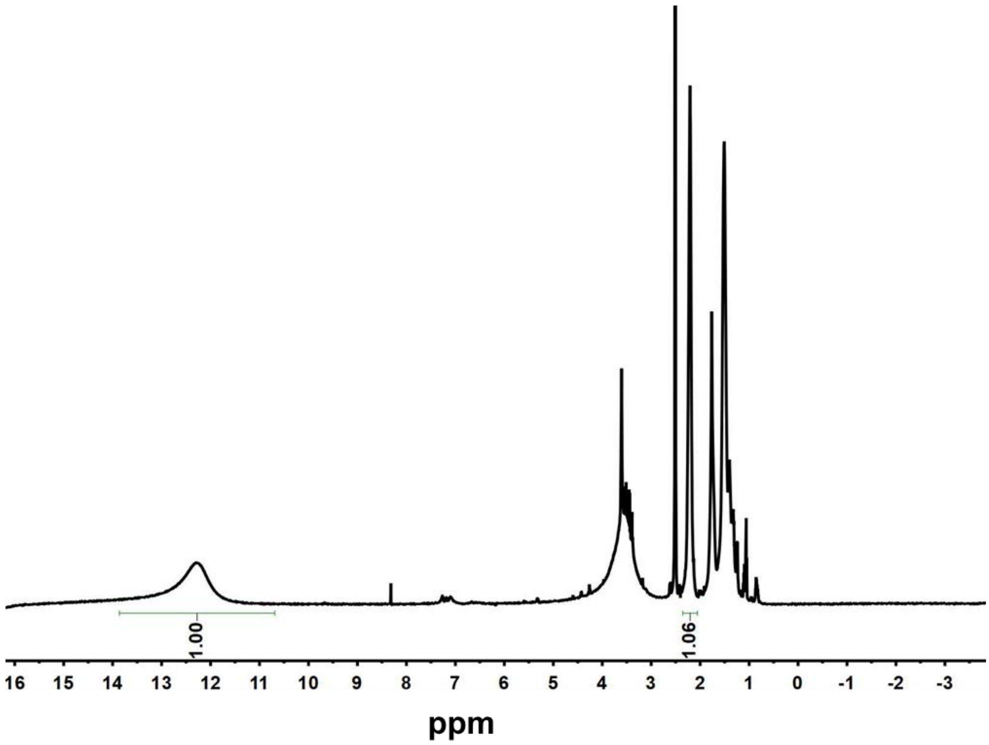

Figure S6 ^{1}H NMR spectra of SAS showing that the deprotection reaction was completed.

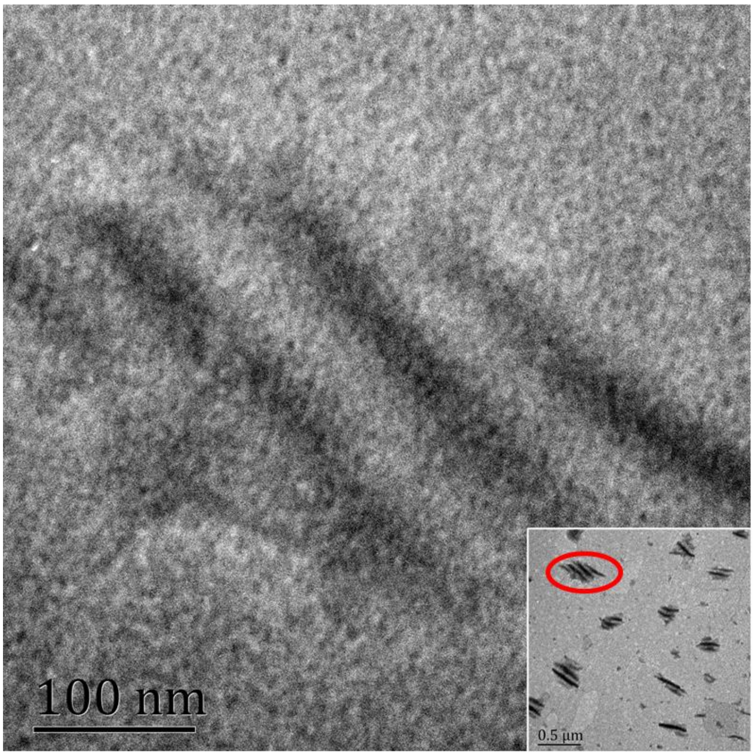

Figure S7 TEM image of thin-sectioned SAS/SES strip sample showing the microphase separated lamellar structure. Inset was the zoomed-out view.

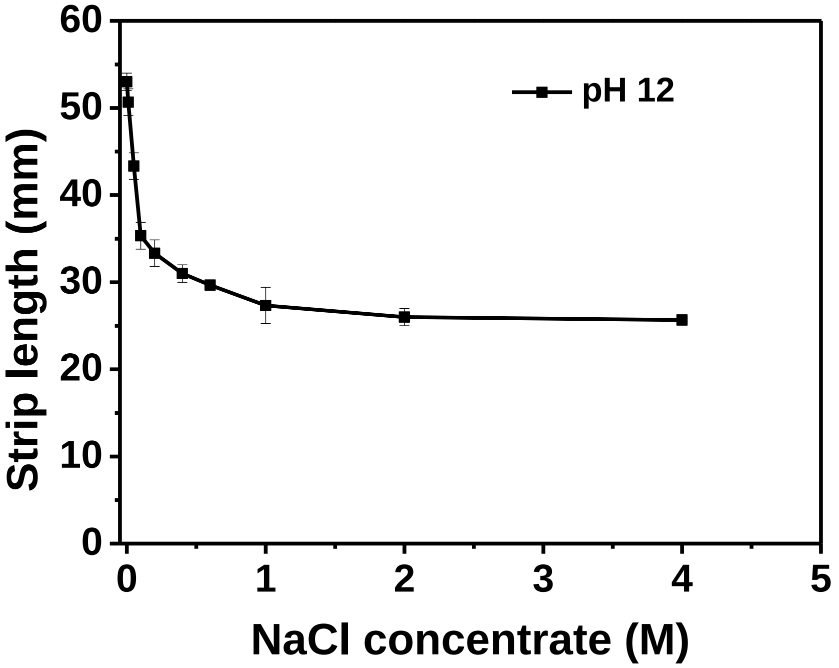

Figure S8 Lengths of the SAS/SES strips immersed in solutions of pH = 12 but different NaCl concentrations.

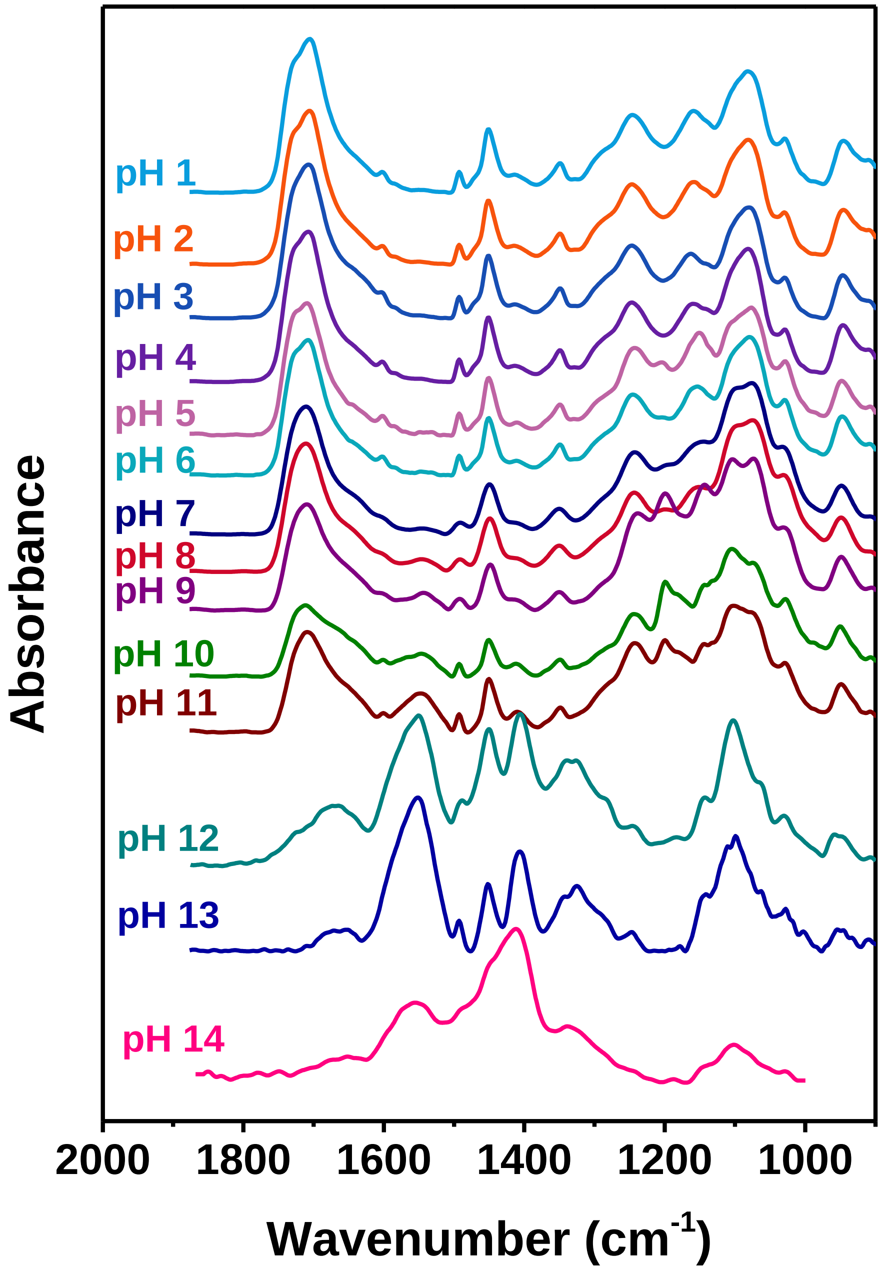

Figure S9 FT-IR spectra of the SAS/SES strips equilibrated in the solutions of different pH values. The strips were soaked in the solutions for more than 10 h and completely dried in a vacuum oven before the measurements.

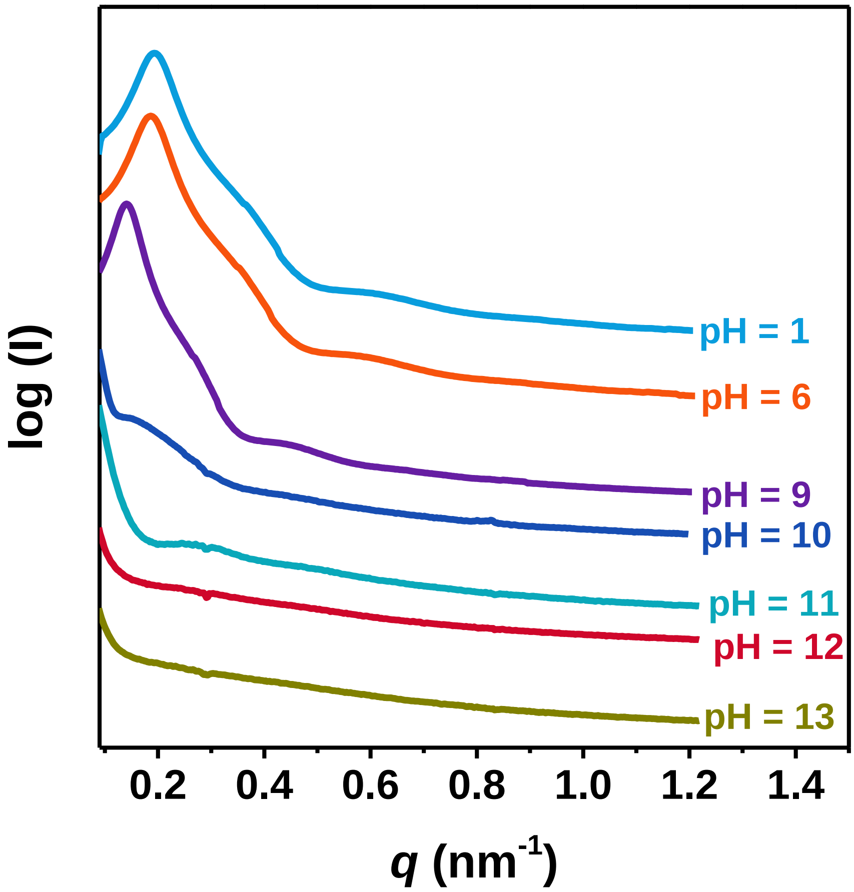

Figure S10 SAXS profiles of the SAS/SES strips equilibrated in the solutions of different pH values. The strips were soaked in the solutions for more than 10 h.

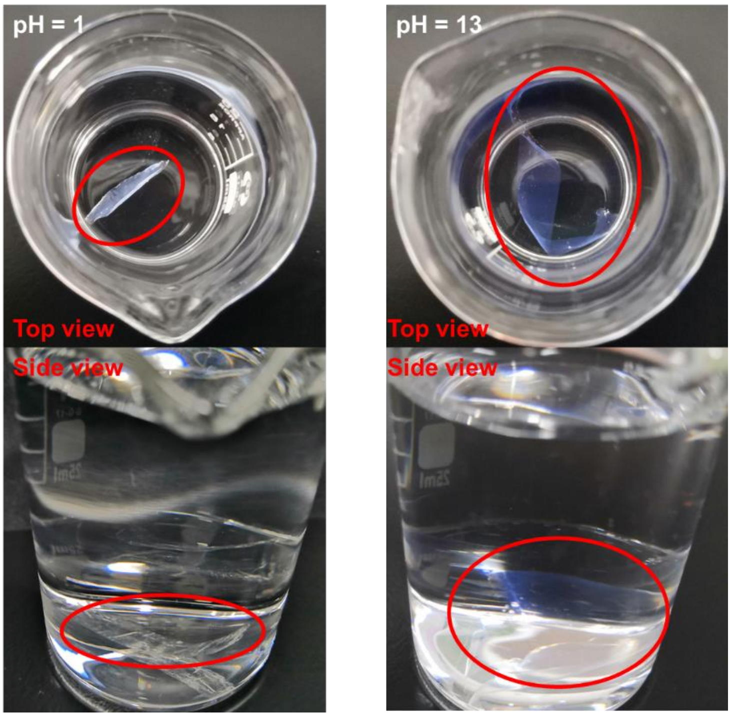

Figure S11 Photos to illustrate the strip will not produce buoyancy and affect its driving performance.

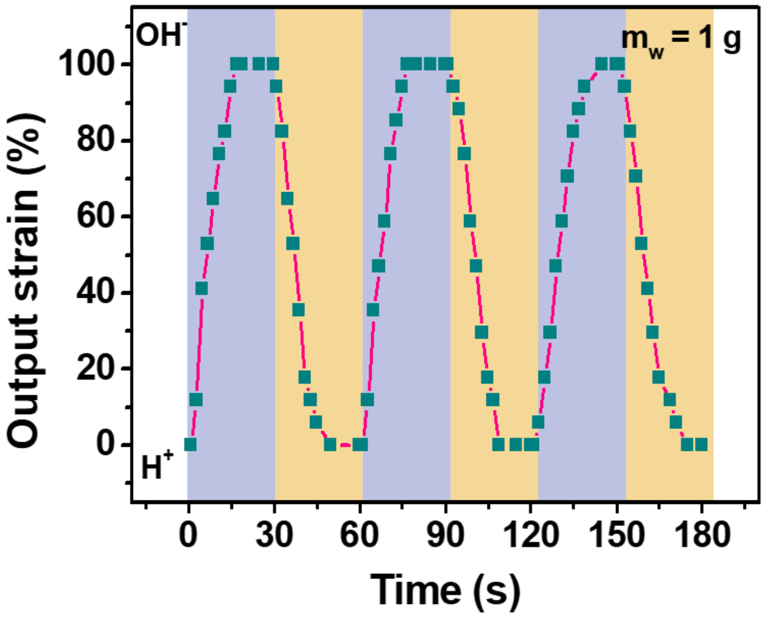

Figure S12 Actuation of the SAS/SES strip with a cargo of 1.0 g when alternately placed in pH = 1 and pH = 13 solutions.

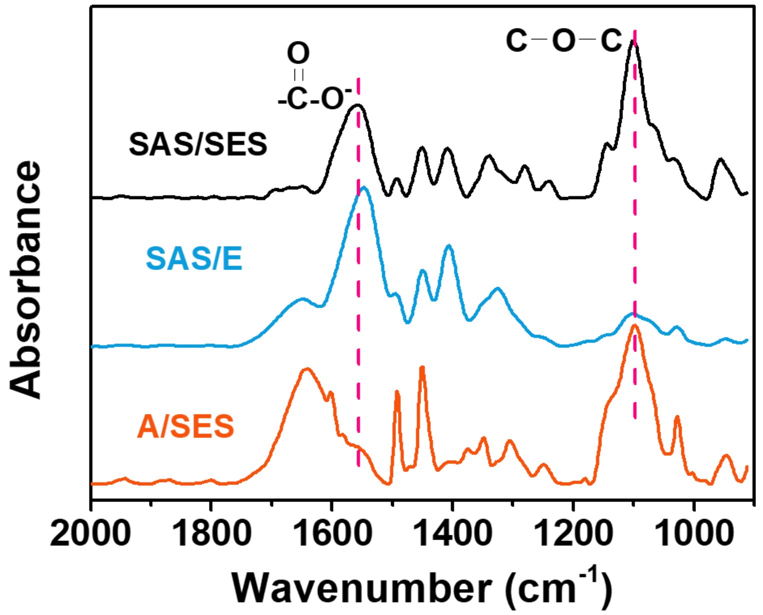

Figure S13 FT-IR spectra of the SAS/SES, SAS/E and A/SES strips equilibrated in solutions with pH = 13. The strips were soaked in the solutions for more than 10 h and completely dried in a vacuum oven before the measurements.

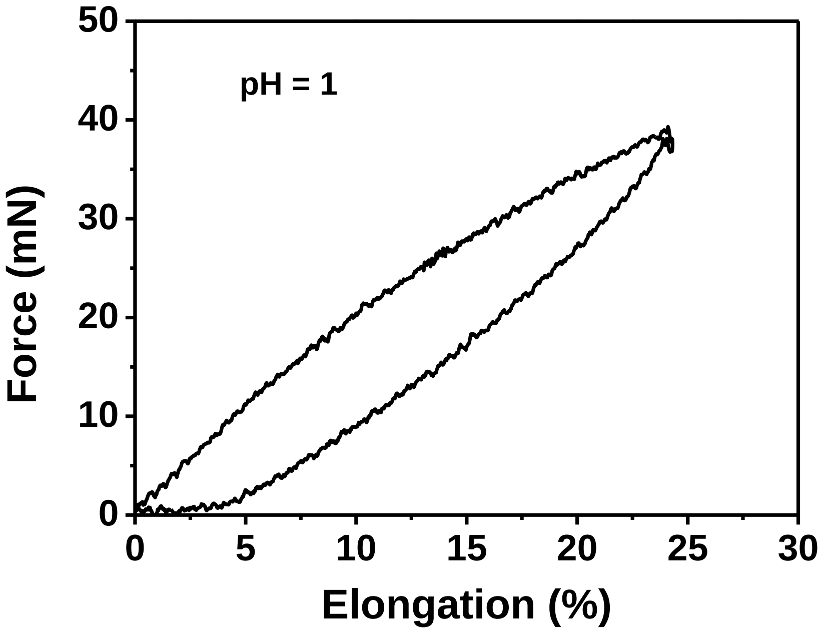

Figure S14 One cyclic stress-strain curve of the SAS/SES strip at pH = 1 showing the hysteresis beyond linear region.

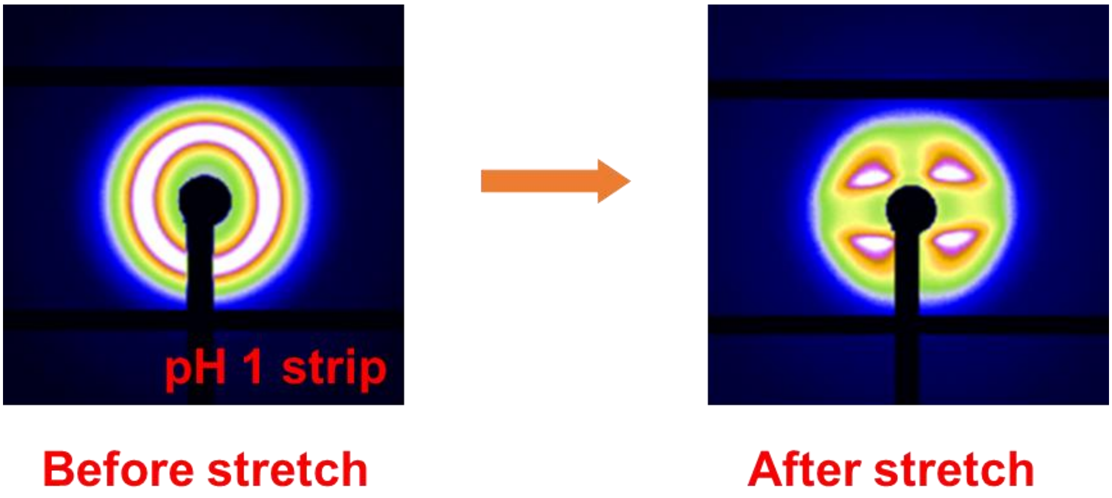

Figure S15 2D SAXS pattern of SAS/SES before and after stretch heavily (beyond linear region).

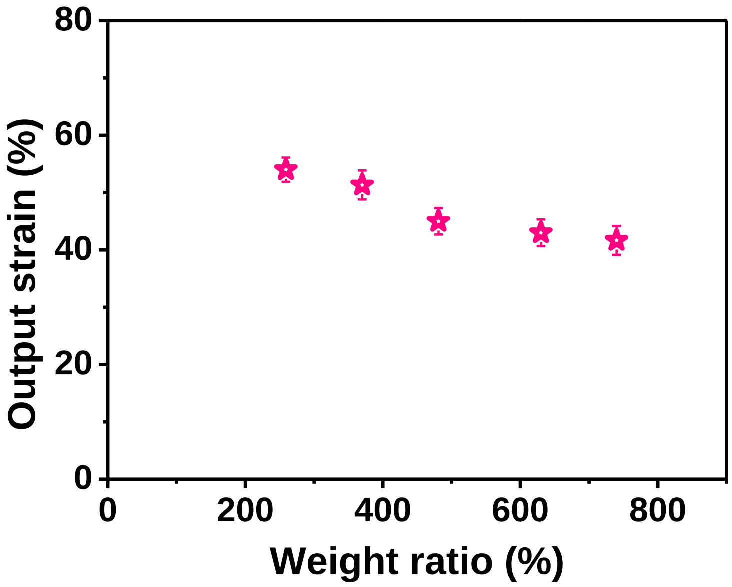

Figure S16 Output strain of the SAS/SES strips determined at different weight ratios.

Reference

[1] J. T. Lai, D. Filla, R. Shea, *Macromolecules* **2002**, *35*, 6754.
[2] W. Zhang, B. Fang, A. Walther, A. H. E. Müller, *Macromolecules* **2009**, *42*, 2563.
